# Herbivory in the soft coral *Sinularia flexibilis* (Alcyoniidae)

**DOI:** 10.1038/srep22679

**Published:** 2016-03-08

**Authors:** Chiara C. Piccinetti, Roberta Ricci, Chiara Pennesi, Giuseppe Radaelli, Cecilia Totti, Alessandra Norici, Mario Giordano, Ike Olivotto

**Affiliations:** 1 Dipartimento di Scienze della Vita e dell’Ambiente, Università Politecnica delle Marche, via Brecce Bianche, 60131 Ancona, Italy; 2 Dipartimento di Biomedicina Comparata e Alimentazione, Università degli Studi di Padova, Agripolis, Viale dell’Università, 16, 35020 Legnaro, Italy; 3 Institute of Microbiology ASCR, Centrum Algatech, Laboratory of photosynthesis, Opatovický mlýn, 379 81 Třeboň, Czech Republic; 4 National Research Council, Institute of Marine Science, Venezia, Italy

## Abstract

Our work provides strong support for the hypothesis that *Sinularia flexibilis* ingests diatoms such as *Thalassiosira pseudonana*. We assessed algal ingestion by *S. flexibilis* through estimates of algal removal, histological analyses, scanning electron microscopy observations, and gene expression determination (18S and silicon transporter 1) by real time PCR. Cell counts are strongly suggestive of algal removal by the coral; light and scanning microscopy provide qualitative evidence for the ingestion of *T. pseudonana* by *S. flexibilis*, while molecular markers did not prove to be sufficiently selective/specific to give clear results. We thus propose that previous instances of inability of corals to ingest algae are reconsidered using different technical approach, before concluding that coral herbivory is not a general feature.

Many corals are capable of both autotrophy, thanks to their photosynthetic symbionts, and heterotrophy^1^,^2^,^3^,^4^. The contribution of heterotrophy to coral nutrition can be substantial in several species and under conditions such as bleaching and low irradiance^3^,^5^,^6^. Coral heterotrophy can be supported by various substrates, including particulate organic matter^5^,^7^, bacteria^8^,^9^,^10^, zooplankton and phytoplankton, including diatoms^1^,^11^,^12^,^13^,^14^,^15^,^16^. Heterotrophy is probably necessary to maintain an appropriate elemental stoichiometry of the animal, provided that the algae typically export to the animal mostly organic C skeletons poor in other nutrients^17^,^18^. For example, in the scleractinian coral *Stylophora pistillata* Esper, heterotrophy increases tissue protein concentration and stimulates growth both directly, by enhancing calcification and organic matrix synthesis, and indirectly, by increasing photosynthetic rates by ameliorating nutrient limitation^3^.

The ability of different coral species to feed on plankton has been studied using different approaches and methodologies, including microscopy, removal rates in feeding tanks, labeling of the prey with radioactive/fluorescent markers, stable isotope analysis^19^ and use of specific sequences to identify ingested preys^15^,^16^,^20^,^21^,^22^,^23^,^24^,^25^,^26^,^27^. Most of these studies have been conducted on zooplankton; the data on phytoplankton grazing by corals are scant, although the relevance of phytoplankton in food webs^28^ and in coral feeding^15^,^23^,^29^,^30^,^31^,^32^,^33^,^34^ is of paramount importance.

The heterotrophic nature of corals varies greatly among species and with respect to morphology, habitats, food availability and symbiosis with zooxanthellae^14^,^23^,^24^. For instance, Sorokin^13^ showed that *Zoanthus sociatus* and *Mopsella auranta* ingest large amounts of microalgae, while no significant ingestion was observed for *Dendronephthya* sp. Farrent and collaborators^27^ observed a very low uptake of algae by *Capnella goboensis*. More recently, Leal and collaborators^24^ reported differences in herbivory in a number of coral species, including the symbiotic soft corals *Heteroxenia fuscescens, Sinularia flexibilis*, the asymbiotic scleractinian coral *Tubastrea coccinea*, the symbiotic scleractinian corals *Stylophora pistillata, Pavona cactu,* and *Oculina arbuscula*. In the study reported by Leal and collaborators^23^ the corals were offered several algal species such as the cryptophyte *Rhodomonas marina*, the haptophytes *Isochrysis galbana* and *Phaeocystis globosa*, and the diatoms *Conticribra weissflogii* and *Thalassiosira pseudonana*. It was suggested that some corals do not ingest algae, while others do. Among the corals that feed on algae, some are selective with respect to the prey, while others may take up other algal species available to them. Furthermore, herbivory is affected by the symbiotic status of the coral (asymbiotic/symbiotic) and, depending on the polyp size. The different behavior of coral species, if confirmed, may have profound implication for their trophic role in reefs. Since the literature comprises a variety of methodological approaches, our aim is to ascertain whether the reported differences in the feeding habits of corals are depending on technical issues.

## Results

### Algal disappearance in the feeding tanks

The mean concentration of algal cells at T_0_ was 1.00 ± 0.1 × 10^5^ cells/mL, in both group B and C. In group B tanks, at T_1_, (p < 0.05) the concentration of *T. pseudonana* cells decreased by over 2 orders of magnitude (2.40 ± 0.3 × 10^3^ cells/mL; Δ_Τ0−Τ1_= 0.98 ± 0.3 × 10^5^ cells/mL) relative to T_0_. In the control tanks (group C), where the coral nubbins were not present, no appreciable change was observed in the concentration of *Thalassiosira* cells at T_1_ (0.97 ± 0.2 × 10^5^ cells/mL; Δ_Τ0−Τ1_=0.03 ± 0.2 × 10^5^ cells/mL).

### Morphological Characteristics

#### Coral surface

**Figure 1:**
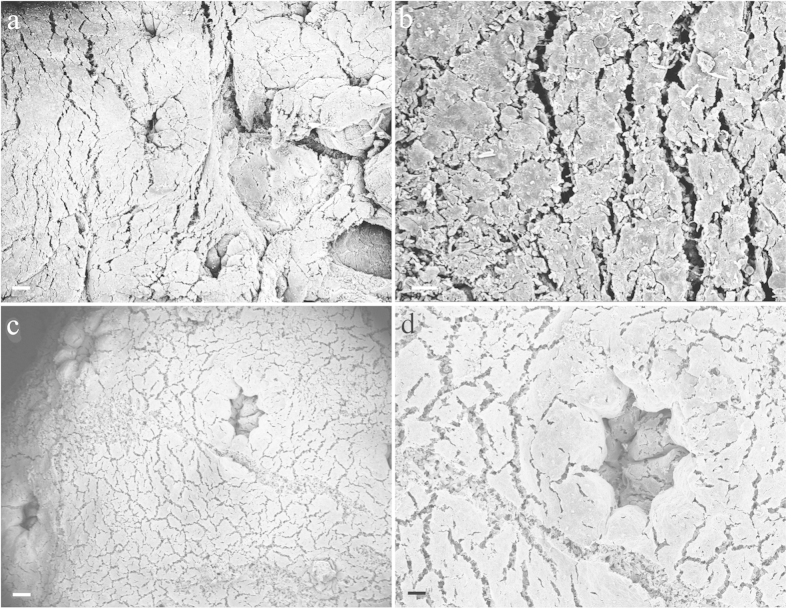
SEM images of coral nubbins. Control (**a,b**) samples (**c,d**) fed with *Thalassiosira pseudonana*. No diatoms were detected on the coral surface. Scale bars: (**a**,**c**) 100 μm; (**b**) 20 μm; (**d**) 50 μm.

Coral samples were sonicated to detach epibionts from their surface and verify if diatoms were present on the surface of the coral nubbins. The results obtained by analysing the sonication water under an optical microscope demonstrated a total absence of *T. pseudonana* and other diatom species. In addition, the SEM analysis of coral nubbins surface revealed that neither *T. pseudonana* nor other diatom species were present on the external surface of the control (group A) (Fig. 1a,b) and fed coral nubbins (group B) (Fig. 1c,d).

#### Study of diatoms inside the coral

**Figure 2:**
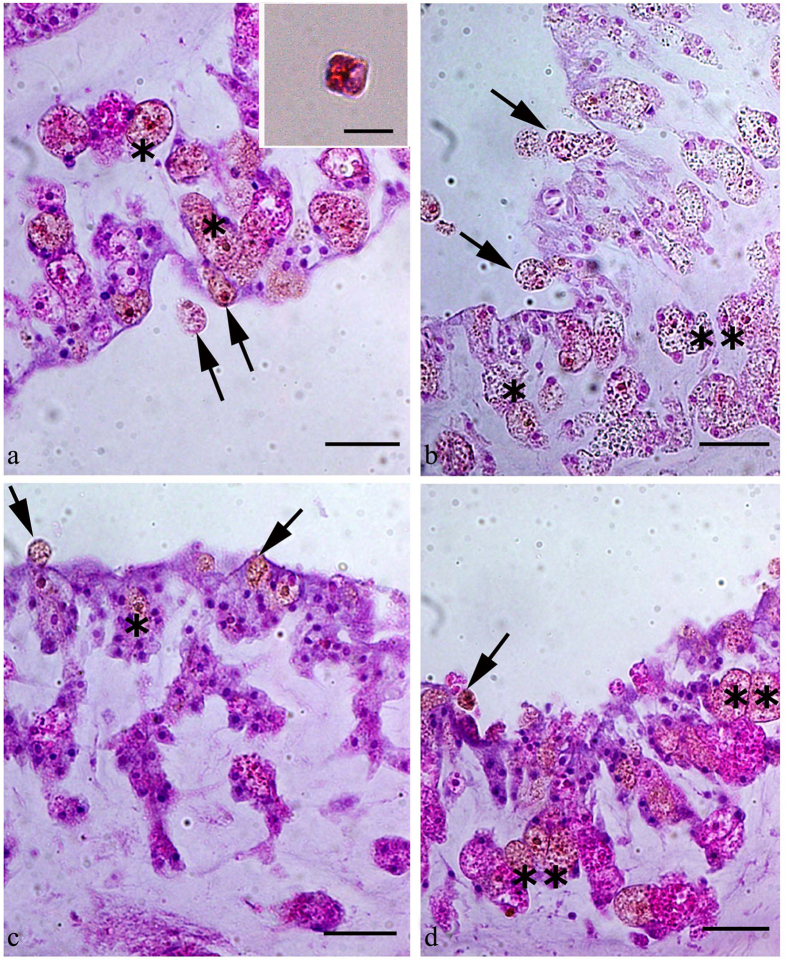
Uptake of the diatom *Thalassiosira pseudonana*by the soft coral *Sinularia flexibilis*. Sections were stained with Mayer’s haematoxylin and eosin. In all panels, the arrows indicate diatom cells in the polyp cavity; *asterisks* indicate diatom cells inside a phagosome in the polyp tissue. Scale bars: (**a**–**d**) 40 μm. The insert in 2A shows a *T. pseudonana* cell stained with Mayer’s haematoxylin.

The histological analysis revealed the presence of *T. pseudonana* only in group B coral samples collected at the end of the feeding experiment. In these samples, the diatoms were detected in the polyp cavity and inside the polyp tissue (Fig. 2a–d). *T. pseudonana* was not detected in group A samples (control) (data not shown), confirming that the coral had not been in contact with *Thalassiosira pseudonana* prior to the experimental feeding.

**Figure 3:**
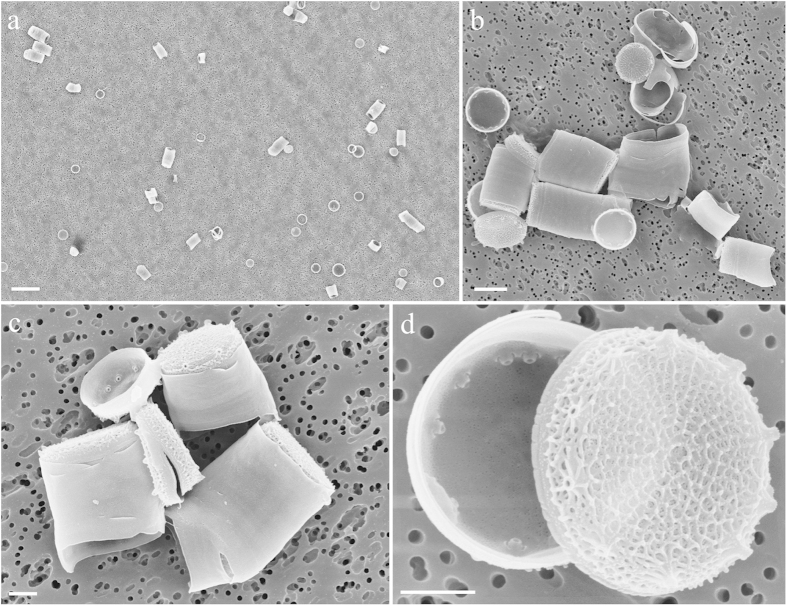
SEM images of *Thalassiosira pseudonana* valves inside the coral, after acid digestion. Scale bars: (**a**) 10 μm; (**b**) 2 μm; (**c**,**d**) 1 μm.

**Figure 4:**
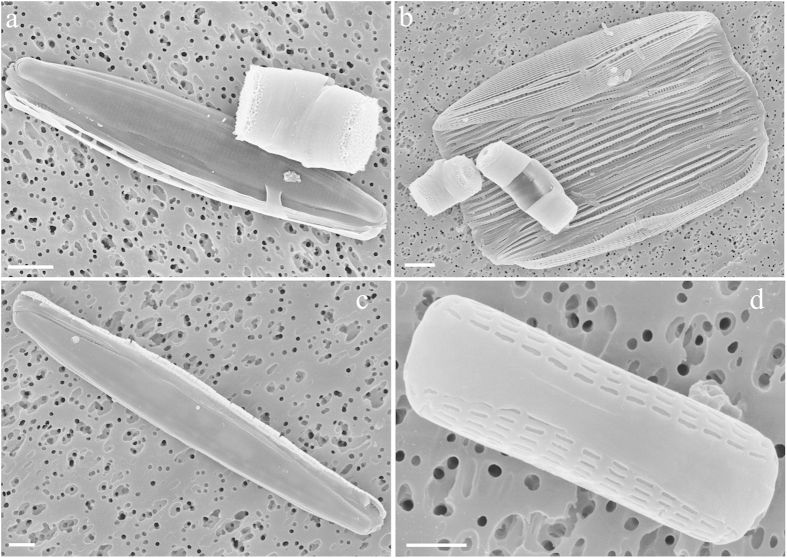
SEM images of benthic diatoms found in the coral, after acid digestion. (**a**) *Nitzschia* sp.; (**b**) *Proshkinia* sp.; (**c**) *Tabularia* sp.; (**d**) *Navicula* sp. Scale bars: (**a**–**c**) 2 μm; (**d**) 1 μm.

After acid digestion of the coral, the SEM analysis of group B samples revealed release of abundant *T. pseudonana* frustules within the coral (Fig. 3a–d). In contrast, we did not find *T. pseudonana* frustules in the control (group A). However, further SEM analyses revealed the presence of other diatoms in both group A and B corals, including *Nitzschia* sp, *Proshkinia* sp, *Tabularia* sp., and *Navicula* sp (Fig. 4). It is possible that these algae were ingested by *S. flexibilis* during rearing in the tanks (Fig. 4a–d).

### Real Time PCR

The specificity of the primers designed from the 18S and the SIT1 silicon transporter sequences of *T. pseudonana* was tested on genomic DNA (gDNA) obtained from pure material of the same alga from a culture collection. We observed a single peak with no primer-dimer formation in all PCR reactions. We further confirmed the amplified product by sequencing and verified the sequence identity by comparison with the sequences deposited in Genbank. PCRs were run using different template concentrations (5, 50 and 500 ng DNA). In coral tissue, amplification of both sequences (18S and SIT1) was always observed. Based on the present observations, however, we cannot rule out cross reactivity of the primers used with other species due to the large degree of similarities that exists for these genes among diatoms. In order to explain that, although we were aware of this problem, we chose the 18S sequence because it had been used previously for similar studies and thus offered an ideal comparison with the data in the literature. The SIT1 transporter was instead selected because exclusive of diatoms.

## Discussion

In the present study, we selected *S. flexibilis* because it was reported not to ingest algae using real-time PCR^24^. We fed this coral with the centric diatom *T. pseudonana*, which is a common species in the marine habitats. Our results provide a strong support for the hypothesis that *S. flexibilis* can ingest diatoms such as *T. pseudonana* and possibly other species. The presence of corals in the tanks with algae significantly reduced the abundance of *T. pseudonana* cells. On the contrary, no significant change in the concentration of *T. pseudonana* cells was observed in the absence of coral nubbins. Therefore, it is reasonable to conclude that the coral ingested *Thalassiosira* when supplied with it. Furthermore, the histological analyses demonstrated the formation of phagosomes around *T. pseudonana* cells exclusively in group B coral nubbins, suggesting that *S. flexibilis* fed on the alga and did not simply ingest it. The presence of the alga inside the corals (group B only) was also confirmed by the SEM images.

Planktonic species such as *T. pseudonana*, which does not have an epizoic life style, may passively adhere to the mucilaginous coral surface^35^,^36^. Epizoic interactions with benthic diatoms have also been described in coral species^37^,^38^ and other cnidarians^39^. In our study, however, SEM analysis of coral surface as well as the analysis of the sonicated water under an optical microscope provided no evidence of the presence of diatoms on the coral surface. The lack of microalgae cells on the surface of *S. flexibilis* may be an indication of a very effective ingestion of the preys by the polyps with the absence of “sloppy” feeding. Alternatively, the obvious cleanness of *S. flexibilis* surface may be a consequence of the presence of diterpenes with antibiotic action, whose production by *S. flexibilis* tissues has been previously demonstrated^40^,^41^. Maida and collaborators^42^ also showed that the adhesion of the brown alga *Ectocarpus* sp. and pennate diatoms to the substrate was inhibited by *S. flexibilis* crude extract.

To our surprise, we were able to amplify segments of 18S and silicon transporter, using genomic DNA extracted from both group A and B coral nubbins. This may be due to the presence of few *T. pseudonana* cells that could not be detected with the other methods or to sequence similarity with other diatom species, e.g. *Nitzschia* sp, *Proshkinia* sp, *Tabularia* sp., and *Navicula* sp. Based on the present results we cannot distinguish between these possibilities. It should be noted that contamination by other diatoms is almost unavoidable, considering that *S. flexibilis* is usually maintained in non-axenic aquaria equipped with rocks and ornaments typically colonized by several benthic microorganisms^43^. Overall, the present study gives strong support to the hypothesis that *S. flexibilis* is capable of herbivory, and can specifically ingest cells of the planktonic diatom *T. pseudonana* in addition to other benthic diatoms. This is in contrast to earlier work on the same species^24^ based only on PCR results. It is thus clear that different methodologies may lead to different conclusions and, based on our study, a multidisciplinary approach is necessary to obtain unequivocal results.

## Methods

### Ethics

All procedures involving animals were conducted in accordance with the European law on experimental animals. Corals were kept in the Animal’s Facility at the Università Politecnica delle Marche (Aut N 84/94-A) in accordance with recommendations by Ministero della Salute using guidelines for the Care and Use of Laboratory Animals. However, since no vertebrates were used in the present study no approval from the ethics committee was necessary.

### Experimental organisms

Diatoms are an important component of phytoplankton in all ecosystems and are responsible for a large proportion of marine primary production^44^. The centric diatom *Thalassiosira pseudonana* is an almost ubiquitous species, with a wide distribution that encompasses tropical areas (www.algaebase.org). It was the first diatom whose genome was fully sequenced^45^ and it has been the object of a large number of studies, through which numerous investigative tools where generated and validated^46^,^47^,^48^,^49^,^50^. *T. pseudonana* therefore represents an excellent experimental organism to study phytoplankton grazing by corals. For similar reasons (i.e. the large number of studies and the variety of methods available), we selected the soft coral *Sinularia flexibilis* as a model organism for our study. Specifically, the fact that *Sinularia flexibilis* was found not to be able to ingest phytoplankton makes this soft coral the ideal experimental model to address the question of whether the differences in the reported feeding habits of corals is depending on technical issues.

### Cultures of Thalassiosira pseudonana

The diatom *T. pseudonana* (CCMP1335) was cultured in 250 mL Erlenmeyer flasks containing 150 mL of AMCONA medium^51^. Cultures were maintained at 20 °C, in continuous light, with a photon flux density of 100 μmol /m^2^ /s (400–700 nm). Cell density was maintained between 10^6^ and 10^7^ cells mL^−1^. Growth rate was determined by daily cell counts, using an automatic counter (CASY TT, Innovatis AG, Reutlingen, Germany). All measurements on the algae were conducted on cells in the exponential growth phase.

### Cultures of Sinularia flexibilis

Five *S. flexibilis* specimens were obtained from an Italian importer, “La Casetta in Canadà” (Settimo Torinese, Italy), who imported them from Indonesia. Corals were maintained in a recirculated 300 L tank equipped with mechanical and biological filtration systems, a protein skimmer and a 12:12 L:D photoperiod. Tanks were filled with artificial seawater (Prodac International, Italy). The parameters in the tank were set as follows: salinity 35 ± 1; pH 8.0 ± 0.2; temperature 26 ± 0.5 °C; nitrate 2 ± 1 mg/L; phosphate 0.02 ± 0.01 mg/L; calcium 400 ± 20 mg/L; magnesium 1300 ± 10 mg/L; photon flux density 350 μmol /m^2^ /s. Ammonia and nitrite were not detectable. Ten per cent of the water was changed weekly.

After 3 months of acclimation, coral nubbins (2 ± 0.3 cm) were prepared from 4 *S. flexibilis* specimens, were stabilized on stubs using needles, and maintained for one more month in the main tank, in order to allow tissue recovery. Nubbins were not fed until the feeding experiment was started.

### Experimental design

Experiments were performed in 1 L aquaria equipped with moderate aeration in order to maintain algae in homogeneous suspension. The aquaria were filled with autoclaved and 0.2 μm-filtered artificial seawater (Prodac International, Italy). The chemical-physical parameters were the same described in the previous paragraph.

### The experimental groups were as follows

Group A (control): three 1 L aquaria with 12 coral nubbins each (total 36 coral nubbins obtained from the 4 different coral colonies - three per specimen per tank). The coral nubbins were maintained in the tanks for 4 hrs.

Group B: three 1 L aquaria with 12 coral nubbins each (total 36 coral nubbins obtained from the 4 different coral colonies - three per specimen per tank). The nubbins were maintained in the tanks for 4 hrs. One hour after the coral nubbins were transferred to the 1 L tanks, which was a sufficient time period for the corals to recover from the transfer and open their polyps, *T. pseudonana* was added at a final concentration of 10^5^ cells/mL.

Group C: three 1 L aquaria to which *T. pseudonana* was added at a final concentration of 10^5^ cells/mL, without coral nubbins.

At the end of the 4-hr experiment, group A and B coral nubbins were sampled by cutting them 3 mm from their basis. The exclusion of the basal part decreased the risk of contamination by benthic diatoms that had been transferred to the experimental tanks with the corals. To minimize parental effects, for each measurement, samples were obtained by pooling nubbins from at least three different specimens. In addition, water samples from aquaria B and C were collected at the beginning and at the end of the experiment to check the algal removal rates by the corals.

### Algal removal in feeding aquaria

At the beginning (T_0_) and at the end of the 4-hr feeding experiment (T_1_), 3 × 10 mL water samples were collected from each of the aquaria of group B and C, to determine the number of *T. pseudonana* cells per mL. Ten 1 mL subsamples from each aquaria of both groups were analyzed using an inverted phase contrast microscope (Zeiss Axiovert 135, Carl Zeiss, Oberkochen, Germany). The treatment time (4 h) was chosen because it is long enough to assess ingestion, but also sufficiently short to minimize artifacts due to excretion and degradation of cells. The entrapment of cells in mucus is unlikely since no mucus accumulation was observed. Furthermore, the contribution of mucus to cell removal was negligible in earlier experiments conducted on the same coral and algae species^23^,^24^.

### Microscopy analyses

Microscopy analysis were conducted to provide a qualitative confirmation of surface contamination and/or presence of algae inside the animal.

#### Coral surface

In order to assess whether *T. pseudonana* was present on the coral surface, 9 nubbins for each experimental group (A and B) were treated as follows: samples were dehydrated by immersion in alcohol at increasing gradations (10, 30, 50, 70, 80, 90, 95 and 100%) and processed through critical point drying (Polaron CPD7501, Quorum Technologies, Newhaven, UK). Then, each coral piece was placed on a biadhesive tape mounted on a stub and sputtered with gold-palladium (Polaron SC7640 Sputter Coater, Quorum Technologies, Newhaven, UK) for observation under a field emission scanning electron microscopy FE SEM (Zeiss Supra 40, Carl Zeiss, Oberkochen, Germany).

#### Thalassiosira pseudonana in the coral

In order to assess the presence of *T. pseudonana* cells in the coral tissues, a histological study was conducted: 9 coral nubbins (3 for each 1 L tank for both group A and B) were fixed by immersion in neutralized 4% paraformaldehyde in phosphate buffer saline (PBS, 0.1 M, pH 7.4), at 4 °C, overnight. The nubbins were subsequently washed in PBS, decalcified (decalcified in 2.5% HCl buffered with 0.1% EDTA), dehydrated through treatment with a graded ethanol series and embedded in paraffin. Then, 4 μm thick consecutive sections were cut using a microtome (Leica RM 2235, Milano, Italy), stained with Mayer’s hematoxylin-eosin, dehydrated, mounted in Eukitt, and examined under an Olympus Vanox photomicroscope (New York Microscope Company, Hicksville, NY, USA) in order to verify the presence of *T. pseudonana.* In addition, a 1 mL sample containing 10^5^ cells/mL of *Thalassiosira pseudonana* was centrifuged at 1500 g for 8 min and the culture media was carefully removed using a pipette. Then the cells were stained with Mayer’s hematoxylin-eosin for 60 s, centrifuged again at 1500 g for 8 minutes to remove the Mayer’s hematoxylin-eosin. Cells were then washed twice in AMCONA media (1500 g, 8 min). After centrifugation the algal pellet was resuspended in 20 μl of AMCONA medium. Finally, the stained and washed sample was observed under an Olympus Vanox photomicroscope (New York Microscope Company, Hicksville, NY, USA) and photographed.

Nine additional coral nubbins (for both group A and B) were sonicated (3 × 10 min, 42 kHz) using an ultrasonic bath (Branson 3510E-DTH, Mexico) in autoclaved/filtered seawater to remove diatoms potentially present on the coral surface. The water obtained after coral sonication, were analyzed with a Zeiss Axiovert 135 inverted light microscope (Carl Zeiss, Oberkochen, Germany) equipped with phase contrast optics to determine if *T. pseudonana* was present; if cells were detected, they were counted.

After sonication, all 9 coral nubbins were treated with nitric and sulphuric acid (1:4 v/v) to remove the organic cell components and allow a proper visualization of the diagnostic features of the frustule. The nubbins were then rinsed several times with distilled water^37^,^52^. The acid-treated samples were analyzed both in light microscopy and at FE SEM in order to check for the presence of *T. pseudonana* valves. For the analysis in light microscopy, samples were mounted on permanent slides with Naphrax^®^ and analyzed with a Zeiss Axioskop light microscope equipped with phase contrast (Carl Zeiss, Oberkochen, Germany). For SEM analysis, samples were air-dried on 0.2 μm polycarbonate membrane filters, mounted with biadhesive tape on aluminum stubs and coated with gold with a sputter coater (Polaron SC7640 Sputter Coater, Quorum Technologies, Newhaven, UK) prior to be examined at FE SEM (Zeiss Supra 40, Carl Zeiss, Oberkochen, Germany). A total of 27 stubs were analyzed.

### DNA extraction, primer design and Real Time PCR

From both group A and B, nine coral nubbins were collected and stored in liquid nitrogen, until gDNA extraction was performed. The extraction was conducted with an Ultraprep miniprep DNA Kit (Microtech, Pozzuoli, Italy) following the manufacter’s instructions. The gDNA was also extracted from *T. pseudonana* CCMP1335. Algae were harvested by centrifugation (1800 g, 8 min). The algal pellet was washed twice in AMCONA medium and centrifuged again (1800 g, 8 min), after which the algal pellet was used for gDNA extraction.This gDNA was used as positive control. The unfed coral nubbins (group A) were used as negative control.

In order to detect the presence of *T. pseudonana* in the coral samples, two sets of specific primers were used; the first pair was used to allow a direct comparison with the data obtained by Leal and collaborators^23^; the second pair was designed using the software package Primer 3 on the *T. pseudonana* sequence available in GenBank:

- (1) *18s ribosomal* RNA (GenBank:AJ535169)^18^For 5′-CTATGCCGACTCAGGATTGG-3′Rev 5′-ATGCACCACCACCCATAGAA-3′
- (2) *Silicon transporter1* (*SIT1*) (GenBank: DQ256066.1)For 5′-TTGCCGAGGATGCCTAAACTT-3′Rev 5′-TGACGAGCTACTGCAGGTTCA-3′

The PCR analyses were performed on coral nubbins (group A and B) and *T. pseudonana* gDNA extracts, using the SYBR Green in an iQ5 iCycler thermal cycler (Bio-Rad, Milan, Italy). All reactions were run in triplicates. The reactions were set on a 96-well plate. A mixture of 500, 50 and 5 ng of gDNA, and 5 μL of 2-fold concentrated iQ TM SYBR Green Supermix (Bio-Rad, Milan, Italy) containing SYBR Green as a fluorescent intercalating agent, 0.3 μM of the forward/reverse primers, were used for each sample. For all reactions, the thermal ramp was as follows: 3 min at 95 °C, 45 cycles of 20 s at 95 °C, 20 s at 55 °C and 20 s at 72 °C. Fluorescence was monitored at the end of each cycle. No amplification product was observed in blanks, where the template was PCR grade water; no primer-dimer formation was detected in control templates. Data were analyzed using the iQ5 optical system software version 2.0 (Bio-Rad, Milan, Italy) including Genex Macro iQ5 Conversion and Genex Macro iQ5 files.

### Statistical methods

All quantitative results (algal removal Δ_Τ0−Τ1_) are expressed as mean ± standard deviation (n = 3). The statistical significance of variance was evaluated with one-way ANOVA, using the statistical software package Sigma Stat 3.1 (Systat Software, Chicago, IL, USA). When significant differences were found, a post-hoc Tukey’s test was applied. A p value < 0.05 was regarded as statistically significant.

## Additional Information

**How to cite this article**: Piccinetti, C. C. *et al*. Herbivory in the soft coral *Sinularia flexibilis* (Alcyoniidae). *Sci. Rep.* **6**, 22679; doi: 10.1038/srep22679 (2016).
